# A Rapid Response to Matrix Therapy With RGTA in Severe Epidermolysis Bullosa

**Published:** 2012-10-17

**Authors:** Ali Al Malaq, Denis Barritault

**Affiliations:** ^a^King Faisal Specialist Hospital and Research Center, Riyadh, Saudi Arabia; ^b^University Paris Est-Creteil and OTR3 4 rue Française, Paris, France

## DESCRIPTION

In this letter, we report a case of a child having 4-year old nonhealing leg ulcers with a severe form of epidermolysis bullosa, successfully treated with a new product based on nano-biodegradable polysaccharide engineered to mimic heparan sulfates. This simple treatment, applied twice a week, induced complete healing within a month and has lasted without recurrence for 2 years. Furthermore, the treatment resulted in a rapid pain relief, whereas standard treatments average of 2 hours daily dressing changes with no closure. This product can bring a solution to this devastating and endless sufferance.

## QUESTIONS

1. **What is the pathophysiology of epidermolysis bullosa?**
2. **What are the complications of epidermolysis bullosa?**
3. **What are the conventional treatments of epidermolysis bullosa?**
4. **Can matrix therapy be a solution to close skin ulcers in epidermolysis bullosa?**

## DISCUSSION

### Introduction

Hereditary epidermolysis bullosa is a group of rare connective tissue diseases characterized by recurrent blister formation in the skin and mucosal membranes in response to mechanical trauma. It is inherited in either an autosomal dominant or recessive manner with an overall incidence and prevalence of about 1/20,000 and 1/125,000 live births, respectively.^1^ Patients require extensive, time consuming, and painful daily wound care to protect the denuded skin. Treatments of epidermolysis bullosa remain mainly at preventing complications from blistering. There is currently no curative treatment, only DNA-based prenatal testing in families at risk of recurrence and several ongoing trials based on cell and/or growth factor therapies.

In this letter, we report the case of recessive dystrophic epidermolysis bullosa, Hallopeau-Siemens type, in a 12-year-old child with chronic nonhealing painful lower extremity ulcers that healed within 4 weeks after a twice weekly topical application of a solution of RGTA (OTR3, Paris, France). This product can bring a solution to this devastating and endless sufferance.

### Case description

**Figure 1:**
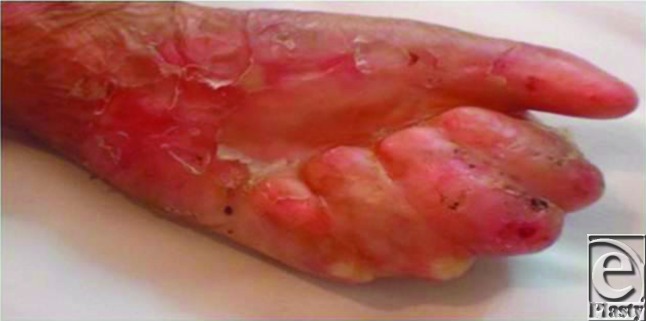
Pseudosyndactyly hand of the 12 years old child, which is nearly always associated to Hallopeau-Siemens type of epidermolysis bullosa.

**Figure 2:**
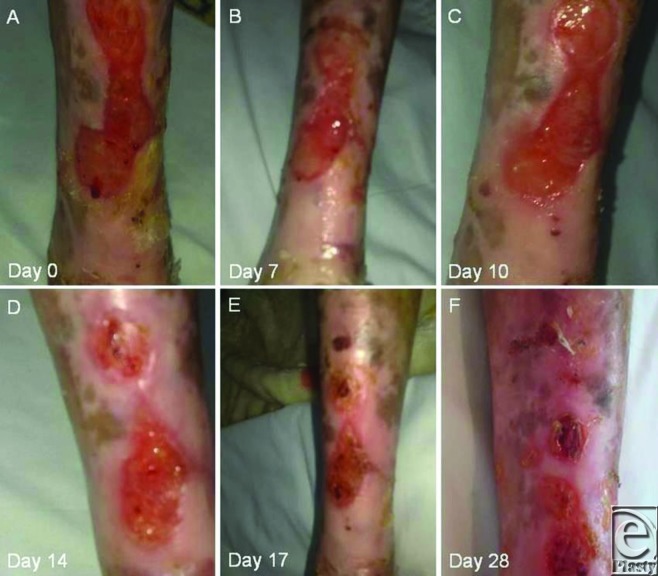
Leg skin color changes and wound area reduction until complete closure during RGTA treatment of a patient with epidermolysis bullosa.

A 12-year-old child with a Hallopeau-Siemens type of epidermolysis bullosa and suffering from nonhealing leg ulcers for 4 years was treated at the clinician's initiative after approval of the parents, with a solution of RGTA (Fig 1). The treatment consisted of topical application of a cotton gauze impregnated with the solution to the wound bed twice weekly for 5 minutes per application, combined with a daily nonadhesive dressing change (without compound). Significant pain relief was obtained within 5 minutes after the first administration of the product. Although intense pain recurred by the next day, it was not as severe as described prior to the treatment. Similar pain relief was observed after each application. After 1 week (2 applications), pain was reduced by 80% and then subsequently disappeared. Skin color changed (Fig 2B) with a decrease in inflammation with subsequent granulation and healing (Fig 2C). After 2 weeks of RGTA application, the wound area was reduced (Fig 2D) until complete closure at 4 weeks with no oozing (Fig 2F). There was no recurrence during an observation period of 2 years.

### Discussion

(1) What is the pathophysiology of epidermolysis bullosa?

The skin is made of 3 layers: the epidermis, the dermis, and then the hypodermis. In healthy individuals, anchoring proteins, such as collagens and laminins, are important for the maintenance, the hook of the basement membrane zone underlying the epithelium, and for preventing the layers from moving independently. In epidermolysis bullosa patients, the epidermis and dermis have altered or lack some of these anchoring proteins that hold them together. As a result, the skin is extremely fragile and minor mechanical action—such as, rubbing, pressure, or friction—can separate the layers of the skin and form blisters and painful sores, comparable to second- or third-degree burns. This lack of anchoring proteins is known to arise from mutations in at least 15 genes, leading to a broad spectrum of diseases: epidermolysis bullosa simplex involving keratin V and XIV, Junctional epidermolysis bullosa involving laminin-5, alpha-6 beta-4 integrin and BPAG2, dystrophic epidermolysis bullosa characterized by mutations in collagen VII gene, and Kindler syndrome involving KIND1.^1^

(2) What are the complications of epidermolysis bullosa?

Epidermolysis bullosa is commonly observed in children from all ethnic origins, with no gender prediction. Its severity ranges from mild, with localized blistering of the hands and feet, to generalized blistering of the skin, sometimes up to 75%, as well as of the oral cavity and injury to many internal organs, which can lead to death between the 20th to 30th years. As a result of chronic blistering of the skin, these patients suffer from anemia, life threatening infection, and chronic infection, including sepsis, and in the most severe cases from the loss of function in the hands and feet by pseudosyndactyly and musculoskeletal contractures or dystrophy. The mucosae can also be affected, leading to eye disorders, periodontal diseases, esophageal and gastrointestinal strictures, possibly causing feeding difficulties, severe malnutrition, and then growth retardation.

As there is no real way to prevent damage from occurring, as it is very aggressive for most of the children who exhibit signs of the disease, their condition can get progressively worse as it increasingly damages their body tissue over time. This can lead to the development of skin cancers in the recessive forms of epidermolysis bullosa, as a result of the chronic damage done to the skin.^2^

(3) What are the conventional treatments of epidermolysis bullosa?

There is currently no satisfactory method for the treatment of epidermolysis bullosa. epidermolysis bullosa patients must maintain a high standard of personal hygiene and skin care to avoid blister formation and infections. They are dealing with daily dressing changes, including cleansing wounds and removing dead skin, local antibiotherapy, and application of fresh dressings, which can be painful and can take several hours.

Researchers are currently focusing their effort on developing products to help blisters heal by engineered skin grafting and to reverse the phenotype of epidermolysis bullosa patients by gene therapy.

(4) Can matrix therapy be a solution to close skin ulcers in epidermolysis bullosa?

RGTA is the acronym of ReGeneraTing Agent and a new therapeutic class of product with a unique mode of action. RGTA is 1-6 alpha polyglucose with substituted carboxymethyl and sulfated groups chain with relative mean MD 80,000D. It is engineered to mimic and replace the destroyed heparan sulfate in the wounded tissue serving as both a matrix element bridging matrix proteins of the extracellular scaffold and storage protection of bioactive peptides including cytokines and growth factors from degradation.^3^ It appears that when introduced to the bed of the chronic wound it restructures the scaffold organization of matrix proteins and allows the newly synthesized cytokines, chemokines, and growth factors secreted by the cells surrounding the wound to create a cellular microenvironment capable of supporting healing.^2^ The wound bed resumes an organization that resembles the original healthy tissue, leading to a healing process reminding of a regeneration as documented in many preclinical studies,^2^ including skin^4^^-^9 and mucosa.^10^^,^^11^

Today few thousands patients are successfully treated with no reported adverse effects and constitute a growing body of evidence to support the efficacy and safety of this new approach documented by case reports, trials,^12^ and ongoing controlled trials. Pain killing activity was also reported^13^ in another similar product developed to treat corneal persistent epithelial defects and ulcers.^14^^,^^15^ Knowing the safety, efficacy, pain relief, and mode of action allowed the attending clinician to make the decision to apply RGTA solution to the child's leg ulcer.

This report is the only known case of treatment of chronic wounds in epidermolysis bullosa with matrix therapy technology. More cases are expected to come after this publication because a randomized controlled trial may become an ethical issue if pain decreases within hours and healing occurs within weeks. It would also be interesting to investigate the effect of RGTA therapy on the complications of epidermolysis bullosa, such as anemia, feeding difficulties, and eye disorders. Furthermore, the mode of action would not depend on the form of epidermolysis bullosa. Thus, RGTA technology addresses a major need in the treatment of this life-threatening disease.

## References

[1] Fine JD (2010). Inherited epidermolysis bullosa. Orphanet J Rare Dis.

[2] Fine JD, Hintner H (2009). Life With Epidermolysis Bullosa: Etiology, Diagnosis, and Multidisciplinary Care and Therapy.

[3] Van Neck J, Tuk B, Barritault D, Tong M (2012). Heparan Sulfate Proteoglycan Mimetics Promote Tissue Regeneration: An Overview, Tissue Regeneration—From Basic Biology to Clinical Application.

[4] Barbier-Chassefiere V, Garcia-Filipe S, Yue XL (2009). Matrix therapy in regenerative medicine: a new approach to chronic wound healing. J Biomed Mater Res A.

[5] Garcia-Filipe S, Barbier-Chassefiere V, Alexakis C (2007). RGTA OTR4120, a heparan sulfate mimetic, is a possible long-term active agent to heal burned skin. J Biomed Mater Res A.

[6] Tong M, Zbinden MM, Hekking IJ, Vermeij M, Barritault D, van Neck JW (2008). RGTA OTR 4120, a heparan sulfate proteoglycan mimetic, increases wound breaking strength and vasodilatory capability in healing rat full-thickness excisional wounds. Wound Repair Regen.

[7] Tong M, Tuk B, Hekking IM, Vermeij M, Barritault D, van Neck JW (2009). Stimulated neovascularization, inflammation resolution and collagen maturation in healing rat cutaneous wounds by a heparan sulfate glycosaminoglycan mimetic, OTR4120. Wound Repair Regen.

[8] Tong M, Tuk B, Hekking IM (2011). Heparan sulfate glycosaminoglycan mimetic improves pressure ulcer healing in a rat model of cutaneous ischemia-reperfusion injury. Wound Repair Regen.

[9] Tong M, Tuk B, Shang P, Hekking IM, Fijneman EMG (2012). Diabetes-Impaired Wound Healing Is Improved by Matrix Therapy with Heparan Sulfate Glycosaminoglycan Mimetic OTR4120 in Rats. Diabetes.

[10] Morvan FO, Baroukh B, Ledoux D (2004). An engineered biopolymer prevents mucositis induced by 5-fluorouracil in hamsters. Am J Pathol.

[11] Mangoni M, Yue X, Morin C (2009). Differential effect triggered by a heparan mimetic of the RGTA family preventing oral mucositis without tumor protection. Int J Radiat Oncol Biol Phys.

[12] Desgranges P, Louissaint T, Allaire E (2011). First clinical pilot study of matrix protection therapy in vascular disease with regenerating agent technology. J Wound Technol.

[13] Groah SL, Libin A, Spungen M (2011). Regenerating matrix-based therapy for chronic wound healing: a prospective within-subject pilot study. Int Wound J.

[14] Chebbi CK, Kichenin K, Amar N (2008). Pilot study of a new matrix therapy agent (RGTA® OTR4120) in treatment-resistant corneal ulcers and corneal dystrophy. J Fr Ophtalmol.

[15] De Monchy I, Labbé A, Pogorzalek N (2012). Management of herpes zoster neurotrophic ulcer using a new matrix therapy agent (RGTA): a case report. J Fr Ophtalmol.

